# Prediction of Uropathogens by Flow Cytometry and Dip-stick Test Results of Urine Through Multivariable Logistic Regression Analysis

**DOI:** 10.1371/journal.pone.0227257

**Published:** 2020-01-07

**Authors:** Akihiro Nakamura, Aya Kohno, Nobuyoshi Noguchi, Kenji Kawa, Yuki Ohno, Masaru Komatsu, Hachiro Yamanishi

**Affiliations:** 1 Department of Clinical Laboratory Science, Faculty of Health Care, Tenri Health Care University, Tenri, Japan; 2 Department of Clinical Bacteriology, Clinical Laboratory Medicine, Tenri Hospital, Tenri, Japan; Northwestern University Feinberg School of Medicine, UNITED STATES

## Abstract

**Purpose:**

Multidrug-resistant Enterobacteriaceae in urinary tract infection (UTI) has spread worldwide; one cause is overuse of broad-spectrum antimicrobial agents such as fluoroquinolone antibacterials. To improve antimicrobial agent administration, this study aimed to calculate a probability prediction formula to predict the organism strain causing UTI in real time from dip-stick testing and flow cytometry.

**Methodology:**

We examined 372 outpatient spot urine samples with observed pyuria and bacteriuria using dip-stick testing and flow cytometry. We performed multiple logistic-regression analysis on the basis of 11 measurement items and BACT scattergram analysis with age and sex as explanatory variables and each strain as the response variable and calculated a probability prediction formula.

**Results:**

The best prediction formula for discrimination of the bacilli group and cocci or polymicrobial group was a model with 5 explanatory variables that included percentage of scattergram dots in an angular area of 0–25° (P<0.001), sex (P<0.001), nitrite (P = 0.002), and ketones (P = 0.133). For a predicted cut-off value of Y = 0.395, sensitivity was 0.867 and specificity was 0.775 (cross-validation group: sensitivity = 0.840, specificity = 0.760). The best prediction formula for *P*. *mirabilis* and other bacilli was a model with percentage of scattergram dots in an angular area of 0–20° (P<0.001) and nitrite (P = 0.090). For a predicted cut-off value of Y = 0.064, sensitivity was 0.889 and specificity was 0.788 (cross-validation group: sensitivity = 1.000, specificity = 0.766).

**Conclusion:**

Simultaneous use of the calculated probability prediction formula with urinalysis results facilitates real-time prediction of organisms causing UTI, thus providing helpful information for empiric therapy.

## Introduction

Urinary tract infection (UTI) is an infectious disease frequently encountered in daily life and is a representative infectious disease that can cause serious sepsis [1]. Also, because Enterobacteriaceae, which produce extended-spectrum β-lactamase (ESBL) and carbapenemase, have recently spread worldwide and have been detected in urine samples at a high rate, proper use of antimicrobial agents in this infectious disease is demanded [2–4].

Currently, identification of the causative organism of UTI by bacterial culture is the gold standard, but such tests require a long testing time, and it is impossible to obtain the test results concurrently with outpatient treatment. Therefore, in most UTIs, antimicrobial therapy is presently performed with the causative organism being unknown. A system in which the causative organism of UTI can be predicted from dip-stick testing and flow cytometry (FCM), which are the initial tools used in the diagnosis of UTI, is needed.

In recent years, progress in urinalysis has been remarkable, and many studies on the identification of pathogenic bacteria of UTI using FCM have been reported [5–7]. The FCM instrument aspirates urine and splits it into two volumes prior to fluorescent dye staining. In the first volume, the sediment stain polymethine stains nucleic acid in the cells whereas in the second volume, only nucleic acids in the bacteria are stained. Therefore, sediment contents such as blood cells, epithelial cells, and casts are detected in the first volume, and only bacteria are detected in the second volume. After staining, the specimen is delivered to the flow cell for particle analysis by use of a red semiconductor laser of 635 nm. The particles are characterized according to impedance, scattering, and fluorescence light from forward- and side-scatter lights. The forward scatter provides information on particle size, and the side scatter provides information on the internal complexity and surface of the particle. In addition, fluorescence intensity provides information on the nucleic acid content of each particle. FCM technology has dramatically improved the diagnostic accuracy of urinalysis and especially UTI screening. However, FCM alone has limitations in its accuracy. Mass spectrometry has been developed, and adding it into the laboratory workflow has also been reported [8], but this modality is expensive and not practical.

The purpose of this study was to calculate a very accurate probability prediction formula to predict the organism strain causing UTI in real time from traditional dip-stick testing and FCM.

## Methods

### Materials

Among fresh outpatient urine samples submitted to the general urinalysis laboratory of Tenri Hospital between October, 2014 and June, 2015, 372 samples (170 men, 202 women) were chosen in which pyuria (> 5-10/HPF) was confirmed and bacteriuria (> 10^4^ CFU/mL) was observed based on bacterial growth in isolated culture testing. Among them, 105 samples (49 men, 56 women) were defined as the cross-validation group, and the remaining 267 samples (121 men, 146 women) were defined as the training group. This study was approved by the ethical committee of Tenri Hospital and Tenri Health Care University (hospital approval no. 899 and university approval no. 115). Informed consent was waived by the institutional review boards owing to the retrospective study design.

### Dip-stick testing and flow cytometry

We used the AUTION HYBRID AU-4050 urine analyzer (ARKRAY Marketing, Inc., Kyoto, Japan) to measure the target samples, and for the dip-stick test, we used AUTION Sticks 10EA (ARKRAY Marketing, Inc.) to qualitatively measure the 8 items of specific gravity, pH, proteins, glucose, ketones, hemoglobin, nitrite and leukocytes esterase, and we used FCM to quantitatively measure the 3 items of erythrocytes, leukocytes and bacteria.

### BACT scattergram analysis by FCM

A BACT (bacteria) scattergram was obtained from FCM (S1 Fig). In brief, the forward scatter of the Y-axis provides information on particle size, and fluorescence intensity of the X-axis provides information on the nucleic acid content of each particle. Moreover, we used a dot number ratio calculation program for area-specific measurement that we originally developed with Microsoft Visual Basic 2012 (S1 Fig) in which we divided the scattergram into 4 angular areas of 0–20° (Area I), 20–25° (Area II), 25–40° (Area III), and ≥40° (Area IV) from the origin in the X-axis direction and calculated the percentage of dots within each area. To evaluate the accuracy of our developed program, we measured *Staphylococcus aureus* ATCC25923 as a representative cocci and *Escherichia coli* ATCC25922 as a representative bacilli 10 times and confirmed the reproducibility of the dot number ratio (S1 Table). After this evaluation, receiver operating characteristic (ROC) analysis was performed on the target urine specimens to differentiate each strain using the values obtained from this program.

### Calculation of a prediction formula for causative organisms of UTI

We performed bivariate analysis and multiple logistic-regression analysis on the basis of the 8 qualitative urinalysis items, 3 FCM measurement items and BACT scattergram analysis with age and sex as explanatory variables and each strain as the response variable and calculated a probability prediction formula using the training group data. Among the explanatory variables, we defined age, specific gravity and pH as real-type variables, sex as a binary variable, and other items as graded variables and performed the analysis (S2 Table). In addition, the calculated prediction formula was verified in the cross-validation group.

### Microbiologic testing

We inoculated 5% sheep blood agar/Drigalski medium with 10 μL of fresh urine using a loop and aerobically cultured each sample at 37°C for 18 to 24 hours. Bacteria with a bacterial content of 10^4^ CFU/mL or more were judged to be the uropathogen, and the bacteria were identified using matrix-assisted laser desorption ionization time-of-flight mass spectrometry (MALDI-TOF MS). We used MALDI Biotyper (Bruker Daltonik, Bremen, Germany) and conducted ethanol-formic acid protein extraction as the pretreatment method [9].

### Statistical analysis

We used StatFlex Ver. 6.0 (Artech Co., Ltd., Osaka, Japan) as the statistical analysis software and set the level of significance at P = 0.05. Cut-off points using ROC analysis were determined using the Youden index method [10]. In addition, we used the stepwise method to select the explanatory variables for multiple logistic regression. The best model obtained by multiple logistic regression was the model with the lowest AIC value [11, 12]. As described above, the prediction formula calculated by the multiple logistic regression underwent cross-validation by the hold-out method using the training group of 267 strains and cross-validation group of 100 strains. We also confirmed the interaction for all of the explanatory variables of the multiple logistic regression. In verification of the interaction, the interaction item multiplied by each explanatory variable was calculated, and the item was inserted as an explanatory variable for multiple logistic regression to verify whether a significant difference (P<0.05) could be shown.

## Results

### Results of strain identification in the target specimens using microbiologic testing

We performed microbiologic testing including urine culture and identification using MALDI-TOF MS. Among the 267 target specimens in the training group, those in which only a single species of bacteria was detected (single-species group) comprised 251 specimens, and those in which 2 or more strains were detected comprised 16 specimens (polymicrobial group). In the single-species group, bacilli were detected in 169 specimens (bacilli group), which included the following bacterial strains: *Escherichia coli*, 121 strains; *Klebsiella* spp., 28 strains; *Proteus mirabilis*, 9 strains; *Pseudomonas aeruginosa*, 3 strains; *Enterobacter* spp., 6 strains; *Citrobacter* spp., 1 strain and *Serratia marcescens*, 1 strain. In contrast, cocci were detected in 82 specimens (cocci group), which included *Enterococcus* spp., 44 strains; *Staphylococcus* spp., 25 strains and *Streptococcus* spp., 13 strains. Besides, among the 105 specimens in the cross-validation group, bacilli were detected in 50 specimens, which included the following bacterial strains: *E*. *coli*, 35 strains; *K*. *pneumoniae*, 8 strains; *P*. *mirabilis*, 3 strains; and *E*. *cloacae*, *E*. *aerogenes*, *K*. *oxytoca* and *P*. *aeruginosa*, 1 strain each. Cocci were detected in 50 specimens, which included *Enterococcus* spp., 28 strains; *Staphylococcus* spp., 14 strains; and *Streptococcus* spp., 8 strains.

### BACT scattergram analysis by FCM

We used the originally developed dot number counting program (S1 Fig, S1 Table) to calculate the ratio of dot numbers in each angular area of the BACT scattergram generated by FCM, and the results of ROC analysis for each group are shown in Table 1. In the BACT scattergram analysis, discrimination characteristics were satisfactory between the bacilli group and the cocci or polymicrobial group, and between the *P*. *mirabilis* group and the other bacilli group. However, the distinctions were indistinguishable between the single-species group and the polymicrobial group and between the polymicrobial group and the cocci group on the BACT scattergram. In addition, it was not possible to distinguish bacterial species other than *P*. *mirabilis*.

**Table 1:** Results of receiver operating characteristic curve analysis using BACT scattergram analysis.

Discrimination group	Optimal condition	AUC	SE
Single-species group and polymicrobial group	Area IV/all areas	0.7256	0.0584
Cocci group and polymicrobial group	Area III/all areas	0.6543	0.0673
Bacilli group and cocci or polymicrobial group	Area I + II/all areas	0.8414	0.0252
*P*. *mirabilis* group and other bacilli group	Area I/I + II + III	0.8410	0.0461

BACT, bacteria; AUC, area under the curve; SE, standard error.

### Calculation of a prediction formula for causative organisms of UTI

Based on the results of the above-mentioned BACT scattergram analysis, it was possible to discriminate between the bacilli group and the cocci or polymicrobial group and between the *P*. *mirabilis* group and the other bacilli group in the bacilli groups. The analysis results of both comparisons using the other testing items including dip-stick testing are shown below.

#### Differentiation between the bacilli group and cocci or polymicrobial group

The results of the bivariate analysis in the discrimination of the bacilli group and cocci or polymicrobial group are shown in S3 Table, and those of the multivariable logistic regression analysis are shown in Table 2. As a result of the bivariate analysis of the 8 qualitative urinalysis items, 4 FCM measurement items, and age and sex, the items showing P<0.05 were sex (P<0.001), bacteria count (P<0.001), BACT scattergram (P<0.001), specific gravity (P = 0.030), proteins (P = 0.004), ketones (P = 0.043) and nitrite (P<0.001). Moreover, we performed a multivariable logistic regression analysis for which the explanatory variables were selected using the stepwise method, and several models were calculated for the selected variables based on the results of bivariate analysis. As a result of the multivariable logistic regression analysis of these items using response variables to differentiate between the bacilli group and cocci or polymicrobial group, the best final model with the lowest Akaike’s Information Criterion (AIC) value was model 1 that included Area I+II/all areas of the BACT scattergram analysis (P<0.001), sex (P<0.001), nitrite (P = 0.002) and ketones (P = 0.133). The explanatory variables selected in this model were only those selected by the stepwise method. Moreover, we confirmed the interaction for all of the explanatory variables. As a result, interaction was recognized only between nitrite and specific gravity in model 3. However, model 3 including nitrite and specific gravity was not the final model.

**Table 2:** Results of final regression model in the discrimination of bacilli group and cocci or polymicrobial group.

Models	Variables	Regression coefficient β	SE (β)	P value	OR (95% CI)	AIC	AUC (95% CI)
Model 1 (Final model using stepwise method)	α (constant)	3.331	0.472	-	-	238.3	0.875 (0.832–0.917)
	Area I+II/all areas	-5.699	0.762	**<0.001**	0.00335 (0.00075–0.01491)		
	Sex	-1.126	0.335	**<0.001**	0.32449 (0.16836–0.62540)		
	Nitrite	-0.446	0.140	**0.002**	0.64025 (0.48651–0.84258)		
	Ketones	1.686	1.123	0.133	5.40018 (0.59732–48.8210)		
Model 2	α (constant)	3.482	0.523	-	-	239.8	0.874 (0.829–0.917)
	Area I+II/all areas	-5.826	0.786	**<0.001**	0.00295 (0.00063–0.01377)		
	Sex	-1.129	0.335	**<0.001**	0.32345 (0.16759–0.62423)		
	Nitrite	-0.444	0.140	**0.002**	0.64124 (0.48733–0.84375)		
	Ketones	1.838	1.162	0.114	6.28594 (0.64491–61.2690)		
	Proteins	-0.056	0.079	0.481	0.94606 (0.81076–1.10393)		
Model 3	α (constant)	-15.988	28.016	-	-	239.9	0.874 (0.831–0.917)
	Area I+II/all areas	-5.676	0.762	**<0.001**	0.00343 (0.00077–0.01527)		
	Sex	-1.095	0.337	**0.001**	0.33448 (0.17263–0.64810)		
	Nitrite^1^	-0.441	0.140	**0.002**	0.64316 (0.48908–0.84577)		
	Ketones	1.520	1.131	0.179	4.57332 (0.49793–42.0046)		
	Specific gravity^1^	19.053	27.638	0.491	1.881E8 (0.00000–6.30E31)		
Model 4	α (constant)	3.118	0.624	-	-	240.1	0.874 (0.830–0.917)
	Area I+II/all areas	-5.751	0.770	**<0.001**	0.00318 (0.00070–0.01439)		
	Sex	-1.135	0.336	**<0.001**	0.32133 (0.16641–0.62047)		
	Nitrite	-0.492	0.167	**0.003**	0.61131 (0.44046–0.84843)		
	Ketones	1.596	1.120	0.154	4.93138 (0.54932–44.2706)		
	Bacteria count	0.104	0.203	0.512	1.10985 (0.74499–1.65340)		

SE, standard error; OR, odds ratio; CI, confidence interval; AIC, Akaike’s Information Criterion; AUC, area under the curve.

^1^ Nitrite interacts with specific gravity.

We determined model 1 to be the final model to predict between the bacilli group and cocci or polymicrobial group, and the formula was calculated as follows:

$$Y=1/\{1+e^{-(\begin{array}{c} 3.331-5.699\times ratio\;of\;area\;of\;I\;and\;II\;and\;all\;area-1.126\times sex-0.446\times nitrite\\ +1.686\times ketones \end{array})}\}.$$

**Fig 1:**
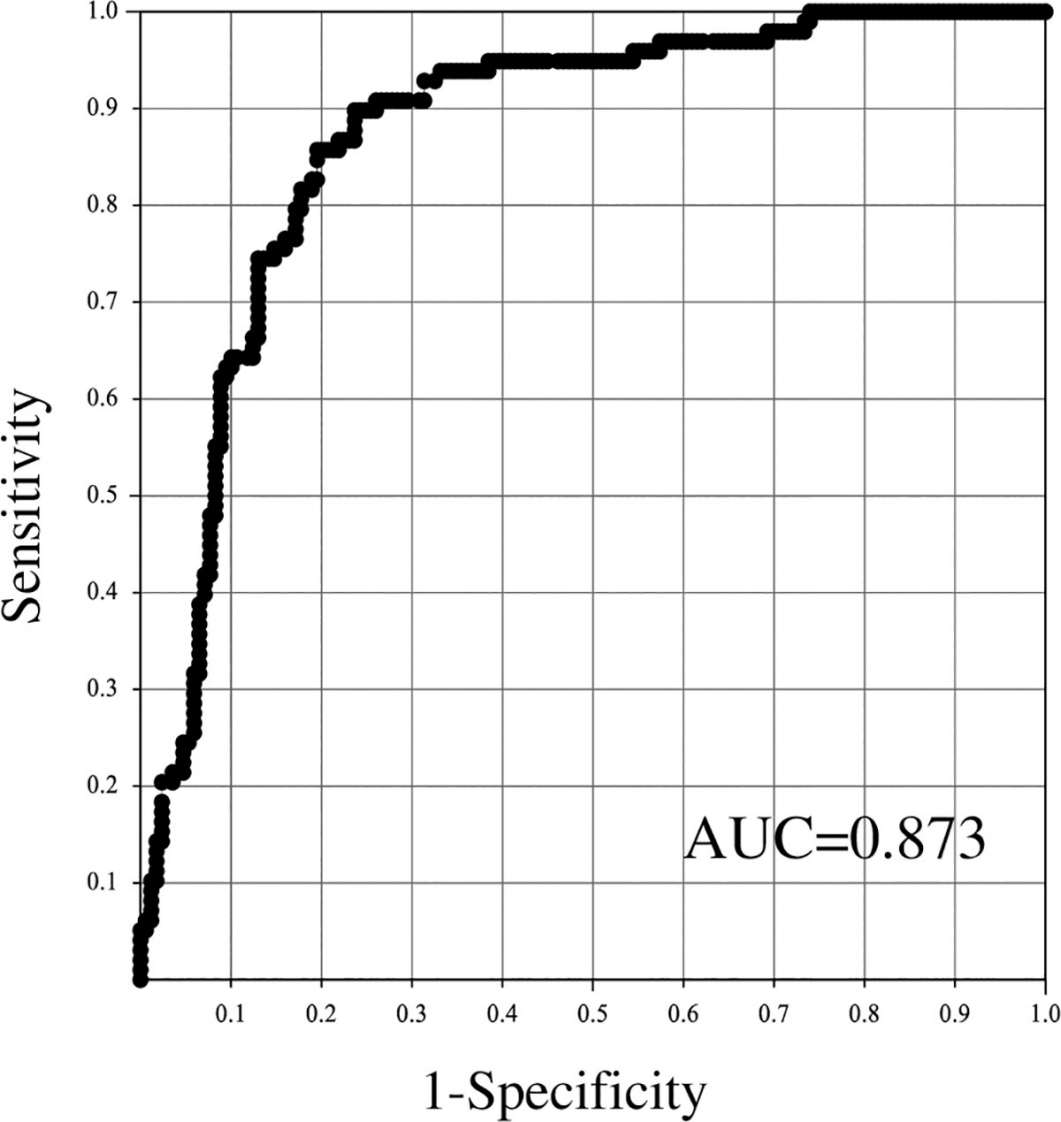
ROC analysis of Y value of the prediction formula to distinguish the cocci or polymicrobial group and bacilli group.

ROC analysis of the predicted Y value is shown in Fig 1, and the cut-off value, sensitivity and specificity are shown in Table 3. The AUC value of this ROC analysis was 0.875. When the cut-off value for the predicted Y value was 0.395, the sensitivity was 0.867 and the specificity was 0.775. In addition, in the cross-validation group, the sensitivity was 0.840 and the specificity was 0.760.

**Table 3:** Sensitivity and specificity in discrimination of bacilli group and cocci or polymicrobial group.

Prediction value	Training group		Validation group	
	Cocci or polymicrobial (n = 98)	Bacilli (n = 169)	Cocci or polymicrobial (n = 50)	Bacilli (n = 50)
Y >0.395	85	38	42	12
Y ≤0.395	13	131	8	38
Sensitivity	0.867		0.840	
Specificity	0.775		0.760	
PPV	0.691		0.778	
NPV	0.910		0.826	

PPV, positive predictive value; NPV, negative predictive value.

#### Differentiation between *P*. *mirabilis* group and other bacilli group among the bacilli groups

Final regression analysis using the *P*. *mirabilis* group and other bacilli groups as the response variables revealed that model 1 shown in Table 4 had the best AIC value. This model with the lowest AIC value included Area I/I+II+III of the BACT scattergram analysis (P<0.001) and nitrite (P = 0.090). Moreover, we confirmed the interaction of all explanatory variables, but it was not. We assumed this model to be the model for calculation of the probability prediction formula to differentiate between the *P*. *mirabilis* group and other bacilli groups and calculated the formula as follows:

$$Y=1/\{1+e^{-(-10.993+11.332\times ratio\;of\;area\;of\;I\;and\;area\;of\;I,II\;and\;III-0.671\times nitrite)}\}.$$

**Table 4:** Results of final regression model in the discrimination of the *P*. *mirabilis* group and other bacilli group.

Models	Variables	Regression coefficient β	SE (β)	P value	OR (95% CI)	AIC	AUC (95% CI)
Model 1 (Final model using stepwise method)	α	-10.993	3.288	-	-	57.4	0.876 (0.787–0.965)
	Area I/I+II+III	11.332	3.957	**<0.001**	83447.7 (35.7456–1.948E8)		
	Nitrite	-0.671	0.396	0.090	0.51109 (0.23505–1.11127)		
Model 2	α	-10.808	3.354	-	-	58.9	0.884 (0.800–0.967)
	Area I/I+II+III	11.196	4.032	**0.006**	72844.3 (26.9418–1.970E8)		
	Nitrite	-0.647	0.401	0.106	0.52365 (0.23883–1.14812)		
	Erythrocytes	-0.441	0.774	0.568	0.64314 (0.14119–2.92970)		
Model 3	α	-9.284	3.805	-	-	58.9	0.881 (0.798–0.961)
	Area I/I+II+III	11.455	3.877	**0.003**	9413.0 (47.2806–1.885E8)		
	Nitrite	-0.682	0.396	0.085	0.50578 (0.23262–1.09973)		
	Age	-0.026	0.034	0.451	0.97470 (0.91190–1.04181)		
Model 4	α	-11.894	4.142	-	-	59.3	0.871 (0.781–0.961)
	Area I/I+II+III	11.058	3.991	**0.006**	63457.4 (25.4179–1.584E8)		
	Nitrite	-0.682	0.401	0.089	0.50565 (0.23059–1.10882)		
	pH	0.179	0.495	0.718	1.19624 (0.45306–3.15848)		

SE, standard error; OR, odds ratio; CI, confidence interval; AIC, Akaike’s Information Criterion; AUC, area under the curve.

**Fig 2:**
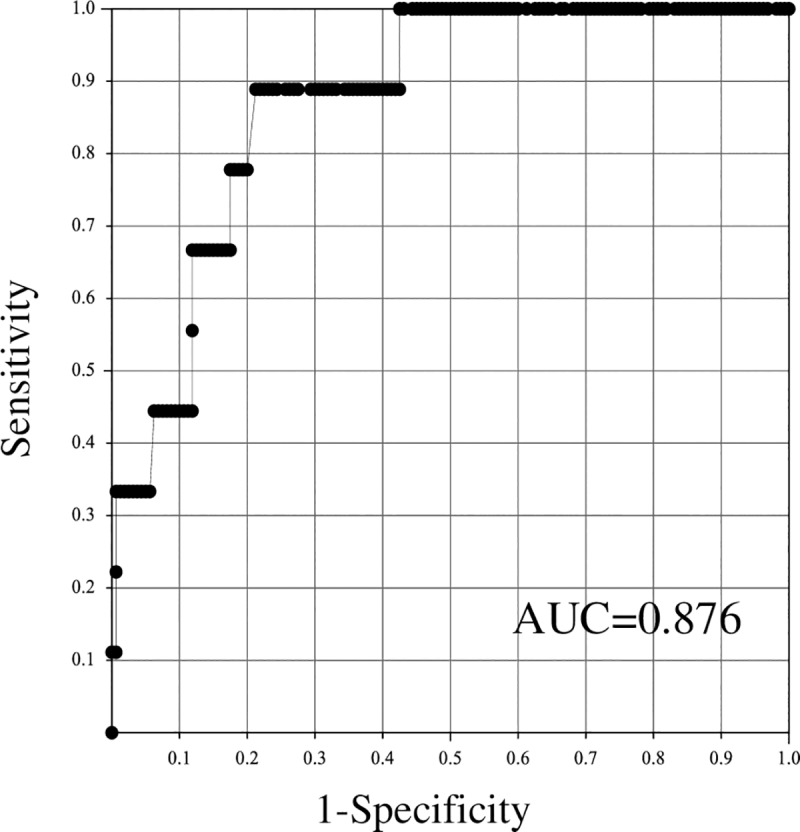
ROC analysis of Y value of the prediction formula to distinguish the *P*. *mirabilis* group and other bacilli group.

ROC analysis of the predicted Y value is shown in Fig 2, and the cut-off value, sensitivity and specificity are shown in Table 5. The AUC value of this ROC analysis was 0.876. When the cut-off value for the predicted Y value was 0.064, the sensitivity was 0.889 and the specificity was 0.788. In addition, in the cross-validation group, the sensitivity was 1.000 and the specificity was 0.766.

**Table 5:** Sensitivity and specificity in discrimination of the *P*. *mirabilis* group and other bacilli group.

Prediction value	Training group		Validation group	
	*P*. *mirabilis* (n = 9)	Other bacilli (n = 160)	*P*. *mirabilis* (n = 3)	Other bacilli (n = 47)
Y >0.064	8	34	3	11
Y ≤0.064	1	126	0	36
Sensitivity	0.889		1.000	
Specificity	0.788		0.766	
PPV	0.191		0.214	
NPV	0.992		1.000	

PPV, positive predictive value; NPV, negative predictive value.

## Discussion

Recently, drug-resistant bacteria including ESBL and carbapenemase-producing Enterobacteriaceae have spread remarkably worldwide [1–4]. These drug-resistant bacteria have been detected in urine samples at high rates, and the use of broad-spectrum antimicrobial agents represented by fluoroquinolone antibacterials is a risk factor [13–16]. To contribute to the appropriate selection of antimicrobial therapy in the treatment of UTI, we devised a predictive formula that can predict causative organisms of UTI using the dip-stick test and FCM, which are the initial tools used in UTI diagnosis.

With regard to the prediction of urinary bacterial strains using FCM, a previous report by Monsen and Rydén found characteristics in each strain of the causative organisms of UTI based on the bacterial count, leucocyte count and red blood cell count, which they calculated using FCM [5]. However, their research only grouped the characteristics data for each causative organism of UTI, and real modeling or verification studies were not conducted. Therefore, it cannot be said that this research is practical as routine work. In addition, because their prediction is classified only on the basis of the 3 items of bacterial count, leucocyte count and red blood cell count, the discrimination ability is limited. We devised a probability prediction formula with higher discrimination characteristics by combining the results of BACT scattergram analysis obtained from FCM with those of qualitative urine analysis.

Furthermore, in a previous report on strain estimation based on scattergram analysis by FCM, Muratani et al. showed that if the angle from the X axis of the approximate curve obtained from the scattergram was less than 30°, the strain was likely to be a Gram-negative bacteria and that if the angle was 30° or more, it was likely to be a Gram-positive bacteria [6, 7]. In the present study, the angle borderline between bacillus and coccus was at 25°. The discrepancy in the results of angle setting of the approximate curve may be due to a difference between the use of a turbid solution of strains in the study of Muratani et al. and the use of clinical urine samples in the present study.

Recent advances in FCM instrumentation have led to the new automated UF-5000 FCM system with higher performance than the device used in the present study [8, 9]. Consequently, a more sophisticated workflow is expected in the future because we can reconstruct a regression equation once through multivariate analysis using this new instrument.

In the BACT scattergram analysis of this study, we determined whether all bacterial species can be distinguished in addition to the distinction between cocci and bacilli using ROC analysis. As a result, it was possible to distinguish between cocci or polymicrobial and bacilli, *P*. *mirabilis* and others, but not other species.

In addition, *P*. *mirabilis* was distinguished only by the results using urine specimens without conducting a pilot study by BACT scattergram analysis using *P*. *mirabilis* ATCC strain because the shape of *P*. *mirabilis* differs when a colony of the strain is measured by FCM and when the urine specimen is measured by FCM. Actually, *P*. *mirabilis* in urine specimens shows a slenderer and longer form on Gram staining than other Enterobacteriaceae, but logarithmic growth colonies do not show such a form. Its shape is similar to that of other Enterobacteriaceae. Therefore, we performed multiple logistic analysis using data from urine specimens to reflect more realistic data from clinical laboratory tests.

We believe that a urinalysis workflow system based on the results of this study will greatly contribute to empirical antimicrobial therapy. According to the Sanford Guide for Infectious Disease Treatment, primary regimens for UTI are trimethoprim-sulfamethoxazole or nitrofurantoin, and alternative regimens are fluoroquinolones such as ciprofloxacin and cephalosporins such as cephalexin [17]. Most of uropathogens are Enterobacteriaceae (approximately 80%), but 10% are *Enterococcus* spp. *Enterococcus* spp. is naturally resistant to trimethoprim-sulfamethoxazole and cephem [18, 19]. Therefore, it is very meaningful to distinguish between bacilli, which are mainly Enterobacteriaceae, and cocci, which are mainly *Enterococcus* spp. Fluoroquinolone or other alternative regimens are effective against both bacilli and cocci, but fluoroquinolone is the most well-recognized risk factor for the emergence of antimicrobial-resistant organisms such as ESBL and carbapenemase-producing Enterobacteriaceae [13–16]. Therefore, this treatment should be avoided whenever possible. Thus, if the workflow system predicts cocci or polymicrobial bacteria, the UTI should be treated with ampicillin rather than trimethoprim-sulfamethoxazole or cephem (and fluoroquinolone should be considered only in cases of severe infectious disease).

Most of the Enterobacteriaceae are *E*. *coli* and *Klebsiella* sp., but they also include *P*. *mirabilis*. UTIs caused by *P*. *mirabilis* may be associated with severe disturbances such as hyperammonemia because it is a urease-producing bacteria [20]. In addition, *P*. *mirabilis* is naturally resistant to first-generation cephem and nitrofurantoin as well as to colistin, which is the first antimicrobial agent for carbapenemase-producing organisms, which are currently feared to spread worldwide. Therefore, if *P*. *mirabilis* is predicted in the workflow system of this study, it is necessary to consider the initial treatment carefully, and it should be treated with trimethoprim-sulfamethoxazole and not nitrofurantoin or cephalexin.

This study has two limitations. First, although this study proved that the prediction formula could roughly classify bacterial groups into the bacilli group and cocci or polymicrobial group, it could not distinguish them according to strain. It could distinguish between the *P*. *mirabilis* group and other bacilli group, but due to the small number of specimens, it will be necessary to increase the number of specimens and perform a re-analysis. Second, we used fresh urine of outpatients suspected of having a UTI as the targeted material for this study, but we did not consider patient backgrounds. Therefore, it is possible that patients such as catheterized patients and pregnant women may have asymptomatic bacteriuria. However, as we usually do not consider the patient’s background in routine urinalysis, the probability prediction equation presented in this study, which does not consider patient background, is optimal when used in daily workflow.

In conclusion, the probability prediction formula calculated in this study could accurately discriminate between bacilli and cocci or multiple species of bacteria as causative organisms of UTI. Incorporating this system into the general urinalysis system may contribute to more appropriate empiric therapy of UTIs. Moreover, there is a likelihood that reducing the use of broad-spectrum antimicrobial agents including fluoroquinolone antibacterials may inhibit the emergence of drug-resistant bacteria.

## Supporting information

S1 Fig The dot number ratio calculation program for area-specific measurement that we originally developed with Microsoft Visual Basic 2012. We divided the scattergram into 4 angular areas of 0–20° (Area I), 20–25° (Area II), 25–40° (Area III), and ≥40° (Area IV) from the origin in the X-axis direction and calculated the percentage of dots within each area. (a) *Escherichia col*i ATCC25922, (b) *Staphylococcus aureus* ATCC25923.
(DOCX)

S1 Table Results of reproducibility testing of the dot number ratio by angular area calculated by the originally developed dot number counting program. (DOCX)

S2 Table Characteristics of all explanatory variables in this study. (DOCX)

S3 Table Bivariate analysis of the distinguishability of the bacilli group and cocci or polymicrobial group using dip-stick testing and flow cytometry. (DOCX)

S4 Table Bivariate analysis of the distinguishability of the *P. mirabilis* group and other bacilli group using dip-stick testing and flow cytometry. (DOCX)

S1 Data (XLS)

## References

[1] Johnson JR. Virulence factors in *Escherichia coli* urinary tract infection. Clin Microbiol Rev 1991; 4: 80–128. 10.1128/cmr.4.1.80 1672263 PMC358180

[2] Paterson DL, Bonomo RA. Extended-spectrum beta-lactamases: a clinical update. Clin Microbiol Rev 2005; 18: 657–86. 10.1128/CMR.18.4.657-686.2005 16223952 PMC1265908

[3] Queenan AM, Bush K. Carbapenemases: the versatile beta-lactamases. Clin Microbiol Rev 2007; 20: 440–58. 10.1128/CMR.00001-07 17630334 PMC1932750

[4] Ohno Y, Nakamura A, Hashimoto E, Matsutani H, Abe N, Fukuda S, et al Molecular epidemiology of carbapenemase-producing Enterobacteriaceae in a primary care hospital in Japan, 2010–2013. J Infect Chemother 2017; 23: 224–9. 10.1016/j.jiac.2016.12.013 28161293

[5] Monsen T, Rydén P. Flow cytometry analysis using sysmex UF-1000i classifies uropathogens based on bacterial, leukocyte, and erythrocyte counts in urine specimens among patients with urinary tract infections. J Clin Microbiol 2015; 53: 539–45. 10.1128/JCM.01974-14 25472486 PMC4298542

[6] Sun SJ, Zuo LL, Liu PP, Wang XM, He ML, Wu SY. The diagnostic performance of urine flow cytometer UF1000i for urinary tract infections. Clin Lab 2018; 64: 1395–401. 10.7754/Clin.Lab.2018.180210 30274017

[7] Muratani T, Kobayashi T, Minamoto Y, Ikuno Y, Migita S. The possibility of the bacterial class estimate using urine from patients with the urinary tract infection by the fully automated urine particle analyzer UF-1000i. Sysmex J Int 2013; 23: 1–9.

[8] De Rosa R, Grosso S, Lorenzi G, Bruschetta G, Camporese A. Evaluation of the new Sysmex UF-5000 fluorescence flow cytometry analyser for ruling out bacterial urinary tract infection and for prediction of Gram negative bacteria in urine cultures. Clin Chim Acta 2018; 484: 171–8. 10.1016/j.cca.2018.05.047 29803898

[9] Nakamura A, Komatsu M, Kondo A, Ohno Y, Kohno H, Nakamura F, et al Rapid detection of B2-ST131 clonal group of extended-spectrum β-lactamase-producing *Escherichia coli* by matrix-assisted laser desorption ionization-time-of-flight mass spectrometry: discovery of a peculiar amino acid substitution in B2-ST131 clonal group. Diagn Microbiol Infect Dis 2015; 83: 237–44. 10.1016/j.diagmicrobio.2015.06.024 26316404

[10] Ruopp MD, Perkins NJ, Whitcomb BW, Schisterman EF. Youden Index and optimal cut-point estimated from observations affected by a lower limit of detection. Biom J 2008; 50: 419–30. 10.1002/bimj.200710415 18435502 PMC2515362

[11] Akaike H. A new look at the statistical model identification. IEEE Transactions on Automatic Control 1974; 19: 716–23.

[12] Vrieze SI. Model selection and psychological theory: a discussion of the differences between the Akaike information criterion (AIC) and the Bayesian information criterion (BIC). Psychol Methods 2012; 17: 228–43. 10.1037/a0027127 22309957 PMC3366160

[13] Kim SY, Park Y, Kim H, Kim J, Koo SH, Kwon GC. Rapid screening of urinary tract infection and discrimination of gram-positive and gram-negative bacteria by automated flow cytometric analysis using Sysmex UF-5000. J Clin Microbiol 2018; 56: pii: e02004–17. 10.1128/JCM.02004-17 29769277 PMC6062802

[14] Wang XH, Zhang G, Fan YY, Yang X, Sui WJ, Lu XX. Direct identification of bacteria causing urinary tract infections by combining matrix-assisted laser desorption ionization-time of flight mass spectrometry with UF-1000i urine flow cytometry. J Microbiol Methods 2013; 92: 231–5. 10.1016/j.mimet.2012.12.016 23305925

[15] Schechner V, Kotlovsky T, Kazma M, Mishali H, Schwartz D, Navon-Venezia S, et al Asymptomatic rectal carriage of *bla*_KPC_ producing carbapenem-resistant *Enterobacteriaceae*: who is prone to become clinically infected? Clin Microbiol Infect 2013; 19: 451–6. 10.1111/j.1469-0691.2012.03888.x 22563800

[16] Ahn JY, Song JE, Kim MH, Choi H, Kim JK, Ann HW, et al Risk factors for the acquisition of carbapenem-resistant *Escherichia coli* at a tertiary care center in South Korea: a matched case-control study. Am J Infect Control 2014; 42: 621–5. 10.1016/j.ajic.2014.02.024 24837112

[17] Sanford Guide for Infectious Disease Treatment 2019 [digital content]. Henry FC. USA.

[18] Matsumoto T, Hamasuna R, Ishikawa K, Takahashi S, Yasuda M, Hayami H, et al Nationwide survey of antibacterial activity against clinical isolates from urinary tract infections in Japan (2008). Int J Antimicrob Agents 2011; 37: 210–8. 10.1016/j.ijantimicag.2010.10.032 21242062

[19] Hayami H, Takahashi S, Ishikawa K, Yasuda M, Yamamoto S, Uehara S, et al Nationwide surveillance of bacterial pathogens from patients with acute uncomplicated cystitis conducted by the Japanese surveillance committee during 2009 and 2010: antimicrobial susceptibility of *Escherichia coli* and *Staphylococcus saprophyticus*. J Infect Chemother 2013; 19: 393–403. 10.1007/s10156-013-0606-9 23640203

[20] Sinha B, Gonzalez R. Hyperammonemia in a boy with obstructive ureterocele and proteus infection. J Urol 1984; 131: 330–1. 10.1016/s0022-5347(17)50366-3 6699967

