# Supplementary material for: Prediction of Uropathogens by Flow Cytometry and Dip-stick Test Results of Urine Through Multivariable Logistic Regression Analysis
Source: PLoS One. 2020 Jan 7;15(1):e0227257. doi: 10.1371/journal.pone.0227257 (PMC6946154; doi:10.1371/journal.pone.0227257)
Supplement: S1 Fig — We divided the scattergram into 4 angular areas of 0–20° (Area I), 20–25° (Area II), 25–40° (Area III), and ≥40° (Area IV) from the origin in the X-axis direction and calculated the percentage of dots within each area. (a) Escherichia coli ATCC25922, (b) Staphylococcus aureus ATCC25923. (DOCX) [file pone.0227257.s001.docx]

**Supplementary Figure 1** The dot number ratio calculation program for area-specific measurement that we originally developed with Microsoft Visual Basic 2012. We divided the scattergram into 4 angular areas of 0-20° (Area I), 20-25° (Area II), 25-40° (Area III), and ≥40° (Area IV) from the origin in the X-axis direction and calculated the percentage of dots within each area. (a) *Escherichia col*i ATCC25922, (b) *Staphylococcus aureus* ATCC25923.
