# Supplementary material for: Prediction of Uropathogens by Flow Cytometry and Dip-stick Test Results of Urine Through Multivariable Logistic Regression Analysis
Source: PLoS One. 2020 Jan 7;15(1):e0227257. doi: 10.1371/journal.pone.0227257 (PMC6946154; doi:10.1371/journal.pone.0227257)
Supplement: S1 Table — (DOCX) [file pone.0227257.s002.docx]

| **Table S1. Results of reproducibility testing of the dot number ratio by angular area calculated by the originally developed dot number counting program.** | | | | | | | | | |
| --- | --- | --- | --- | --- | --- | --- | --- | --- | --- |
| **Assay no.** | ***Staphylococcus aureus* ATCC25923 (%)** | | | |  | ***Escherichia coli* ATCC25922 (%)** | | | |
|  | **Area I** | **Area II** | **Area III** | **Area IV** |  | **Area I** | **Area II** | **Area III** | **Area IV** |
| 1 | 7.8 | 15.9 | 56.9 | 19.4 |  | 38.6 | 28.9 | 29.6 | 3.0 |
| 2 | 8.9 | 18.3 | 57 | 15.8 |  | 39 | 25.8 | 32.3 | 2.9 |
| 3 | 8 | 17.0 | 56.5 | 18.5 |  | 37.9 | 23.7 | 34.5 | 4.0 |
| 4 | 8.5 | 15.1 | 57.6 | 18.9 |  | 39.4 | 27.1 | 30.3 | 3.2 |
| 5 | 7.3 | 15.9 | 58.6 | 18.2 |  | 37.5 | 25.5 | 35.9 | 1.0 |
| 6 | 8.2 | 16.3 | 58.6 | 16.8 |  | 39.4 | 23.7 | 33.8 | 3.2 |
| 7 | 9 | 15.8 | 59 | 16.2 |  | 36.7 | 27.2 | 34.0 | 2.1 |
| 8 | 8.1 | 16.5 | 58.2 | 17.3 |  | 35.8 | 26.7 | 34.5 | 3.0 |
| 9 | 9 | 15.5 | 59 | 16.5 |  | 37.5 | 24.3 | 35.3 | 2.9 |
| 10 | 7.1 | 17.0 | 57.5 | 18.5 |  | 37.7 | 26.9 | 31.9 | 3.6 |
| Mean ± SD | 8.19 ± 0.7 | 16.33 ± 0.9 | 57.89 ± 0.9 | 17.61 ± 1.2 |  | 37.95 ± 1.2 | 25.98 ± 1.7 | 33.21 ± 2.1 | 2.89 ± 0.8 |
| CV (%) | 8.2 | 5.6 | 1.6 | 7.1 |  | 3.1 | 6.6 | 6.3 | 28.6 |
| SD, standard deviation; CV, coefficient of variation. | | | | | | | | | |
