# Supplementary material for: Prediction of Uropathogens by Flow Cytometry and Dip-stick Test Results of Urine Through Multivariable Logistic Regression Analysis
Source: PLoS One. 2020 Jan 7;15(1):e0227257. doi: 10.1371/journal.pone.0227257 (PMC6946154; doi:10.1371/journal.pone.0227257)
Supplement: S2 Table — (DOCX) [file pone.0227257.s003.docx]

| **Table S2. Characteristics of all explanatory variables in this study.** | | | | | | | | | | | |
| --- | --- | --- | --- | --- | --- | --- | --- | --- | --- | --- | --- |
| **Item** | | **Variable type** | **Training group (n=267)** | | | |  | **Cross-validation group (n=100)** | | | |
|  |  |  | **Mean ± SD** | **Median** | **Range** | **Frequency (%)** |  | **Mean ± SD** | **Median** | **Range** | **Frequency (%)** |
| Patient information | Age (years) | Real-type variable | 70.61 ± 14.95 | 74 | 0-95 | - |  | 68.16 ± 17.11 | 72 | 0-91 | - |
|  | Sex (male) | Binary variable^1^ | - | - | **-** | 121 (45.3) |  | - | - | - | 49 (46.7) |
| Flow cytometry | Bacteria count | Graded variable^2^ | 2.88 ± 0.98 | 3 | 0-4 | - |  | 2.78 ± 0.95 | 3 | 0-4 | - |
|  | Leukocytes | Graded variable^3^ | 3.50 ± 1.90 | 4 | 0-6 | - |  | 3.36 ± 1.93 | 4 | 0-6 | - |
|  | Erythrocytes | Graded variable^3^ | 0.67 ± 1.37 | 0 | 0-6 |  |  | 0.86 ± 1.57 | 0 | 0-6 | - |
|  | BACT scattergram  (Area I+II/all areas) | Real-type variable | 0.572 ± 0.261 | 0.602 | 0.046-0.975 | - |  | 0.514 ± 0.268 | 0.470 | 0.061-0.966 | - |
| Dip-stick testing | Specific gravity | Real-type variable | 1.013 ± 0.006 | 1.012 | 1.000-1.035 | - |  | 1.014 ± 0.006 | 1.013 | 1.001-1.031 | - |
|  | pH | Real-type variable | 6.14 ± 0.75 | 6.0 | 5.0-8.0 | - |  | 6.2 ± 0.71 | 6.0 | 5.0-8.0 | - |
|  | Proteins | Graded variable^4^ | 1.48 ± 2.09 | 0 | 0-8 | - |  | 1.73 ± 2.33 | 1 | 0-8 | - |
|  | Glucose | Graded variable^5^ | 0.73 ± 2.10 | 0 | 0-9 | - |  | 0.83 ± 2.31 | 0 | 0-9 | - |
|  | Ketones | Graded variable^6^ | 0.02 ± 0.17 | 0 | 0-2 | - |  | 0.02 ± 0.14 | 0 | 0-1 | - |
|  | Hemoglobin | Graded variable^7^ | 1.57 ± 1.95 | 1 | 0-7 | - |  | 1.69 ± 2.08 | 1 | 0-7 | - |
|  | Nitrite | Graded variable^8^ | 0.97 ± 1.34 | 0 | 0-7 | - |  | 0.83 ± 1.27 | 0 | 0-3 | - |
|  | Leukocytes esterase | Graded variable^9^ | 2.64 ± 1.41 | 3 | 0-4 | - |  | 2.32 ± 1.51 | 2 | 0-4 | - |
| ^1^ 0 for male, 1 for female. ^2^ <10^4/mL to ≥10^7/mL was assigned for rank 0 to 4. ^3^ <5/HPF to ≥100/HPF was assigned for rank 0 to 6. ^4^ Negative to (4+) >1000 mg/dL was assigned for rank 0 to 10. ^5^ Negative to (4+) >1000 mg/dL was assigned for rank 0 to 9. ^6^ Negative to (1+) 20 mg/dL was assigned for rank 0 to 2. ^7^ Negative to (3+) >1.0 mg/dL was assigned for rank 0 to 7. ^8^ Negative to (3+) was assigned for rank 0 to 4. ^9^ Negative to (4+) 500/µL was assigned for rank 0 to 4. | | | | | | | | | | | |
|  |  |  |  |  |  |  |  |  |  |  |  |
|  |  |  |  |  |  |  |  |  |  |  |  |
