# Supplementary material for: Prediction of Uropathogens by Flow Cytometry and Dip-stick Test Results of Urine Through Multivariable Logistic Regression Analysis
Source: PLoS One. 2020 Jan 7;15(1):e0227257. doi: 10.1371/journal.pone.0227257 (PMC6946154; doi:10.1371/journal.pone.0227257)
Supplement: S3 Table — (DOCX) [file pone.0227257.s004.docx]

| **Table S3. Bivariate analysis of the distinguishability of the bacilli group and cocci or polymicrobial group using dip-stick testing and flow cytometry.** | | | | | | | | | | |
| --- | --- | --- | --- | --- | --- | --- | --- | --- | --- | --- |
| **Item** | | **Variable type** | **Mean ± SD or n(%)** | | **P value** | **AUC (95% CI)** | **Cut-off value** | **OR (95% CI)** | **Sensitivity** | **Specificity** |
|  |  |  | **Cocci or polymicrobial group (n=98)** | **Bacilli group (n=169)** |  |  |  |  |  |  |
| Patient information | Age (years) | Real-type variable | 69.15 ± 19.49 | 71.46 ± 11.52 | 0.605 | - | - | - | - | - |
|  | Sex (male) | Binary variable^1^ | 61 (62.24) | 60 (35.50) | **<0.001** | - | - | 2.995 (1.802-4.978) | 0.622 | 0.645 |
| Flow cytometry | Bacteria count | Graded variable^2^ | 2.62 ± 0.91 | 3.02 ± 0.98 | **<0.001** | 0.623 (0.555-0.690) | 3 | 0.297 (0.163-0.541) | 0.163 | 0.604 |
|  | Leukocytes | Graded variable^3^ | 3.59 ± 2.00 | 3.45 ± 1.84 | 0.514 | - | - | - | - | - |
|  | Erythrocytes | Graded variable^3^ | 0.90 ± 1.66 | 0.54 ± 1.16 | 0.131 | - | - | - | - | - |
|  | BACT scattergram  (Area I+II/all areas) | Real-type variable | 0.377 ± 0.2 | 0.686 ± 0.2 | **<0.001** | 0.838 (0.791-0.885) | 0.652 | 19.415 (10.007-37.667) | 0.918 | 0.633 |
| Dip-stick testing | Specific gravity | Real-type variable | 1.014 ± 0.006 | 1.012 ± 0.006 | **0.030** | 0.580 (0.510-0.650) | 1.011 | 2.293 (1.368-3.845) | 0.684 | 0.515 |
|  | pH | Real-type variable | 6.153 ± 0.679 | 6.136 ± 0.786 | 0.680 | - | - | - | - | - |
|  | Proteins | Graded variable^4^ | 1.87 ± 2.17 | 1.25 ± 2.02 | **0.004** | 0.596 (0.526-0.667) | 0 | 2.051 (1.241-3.390) | 0.592 | 0.586 |
|  | Glucose | Graded variable^5^ | 0.85 ± 2.32 | 0.67 ± 1.97 | 0.740 | - | - | - | - | - |
|  | Ketones | Graded variable^6^ | 0.05 ± 0.26 | 0.01 ± 0.08 | **0.043** | 0.517 (0.445-0.590) | 0 | 7.149 (1.068-47.860) | 0.041 | 0.994 |
|  | Hemoglobin | Graded variable^7^ | 1.70 ± 2.06 | 1.50 ± 1.88 | 0.547 | - | - | - | - | - |
|  | Nitrite | Graded variable^8^ | 0.53 ± 1.10 | 1.22 ± 1.40 | **<0.001** | 0.625 (0.558-0.693) | 2 | 2.973 (1.579-5.598) | 0.857 | 0.331 |
|  | Leukocytes esterase | Graded variable^9^ | 2.68 ± 1.42 | 2.61 ± 1.41 | 0.632 | - | - | - | - | - |
| ^1^ 0 for male, 1 for female. ^2^ <10^4/mL to ≥10^7/mL was assigned for rank 0 to 4. ^3^ <5/HPF to ≥100/HPF was assigned for rank 0 to 6. ^4^ Negative to (4+) >1000 mg/dL was assigned for rank 0 to 10. ^5^ Negative to (4+) >1000 mg/dL was assigned for rank 0 to 9. ^6^ Negative to (1+) 20 mg/dL was assigned for rank 0 to 2. ^7^ Negative to (3+) >1.0 mg/dL was assigned for rank 0 to 7.  ^8^ Negative to (3+) was assigned for rank 0 to 4. ^9^ Negative to (4+) 500/µL was assigned for rank 0 to 4. | | | | | | | | | | |
|  |  |  |  |  |  |  |  |  |  |  |
|  |  |  |  |  |  |  |  |  |  |  |
