# Supplementary material for: Prediction of Uropathogens by Flow Cytometry and Dip-stick Test Results of Urine Through Multivariable Logistic Regression Analysis
Source: PLoS One. 2020 Jan 7;15(1):e0227257. doi: 10.1371/journal.pone.0227257 (PMC6946154; doi:10.1371/journal.pone.0227257)
Supplement: S4 Table — (DOCX) [file pone.0227257.s005.docx]

| **Table S4. Bivariate analysis of the distinguishability of the *P. mirabilis* group and other bacilli group using dip-stick testing and flow cytometry.** | | | | | | | | | | |
| --- | --- | --- | --- | --- | --- | --- | --- | --- | --- | --- |
| **Item** | | **Variable type** | **Mean ± SD or n(%)** | | **P value** | **AUC (95% CI)** | **Cut-off value** | **OR (95% CI)** | **Sensitivity** | **Specificity** |
|  |  |  | ***P. mirabilis* group (n=9)** | **Other bacilli group (n=160)** |  |  |  |  |  |  |
| Patient information | Age (years) | Real-type variable | 68.78 ± 14.81 | 71.61 ± 11.35 | 0.232 | - | - | - | - | - |
|  | Sex (male) | Binary variable^1^ | 3 (33.33) | 57 (35.63) | 0.889 | - | - | - | - | - |
| Flow cytometry | Bacteria count | Graded variable^2^ | 2.89 ± 0.60 | 3.03 ± 1.00 | 0.395 | - | - | - | - | - |
|  | Leukocytes | Graded variable^3^ | 3.78 ± 1.79 | 3.43 ± 1.85 | 0.551 | - | - | - | - | - |
|  | Erythrocytes | Graded variable^3^ | 0.11 ± 0.33 | 0.57 ± 1.19 | 0.223 | - | - | - | - | - |
|  | BACT scattergram  (Area I/I+II+III) | Real-type variable | 0.837 ± 0.084 | 0.654 ± 0.168 | **<0.001** | 0.841  (0.751-0.931) | 0.733 | no data | 1.000 | 0.638 |
| Dip-stick testing | Specific gravity | Real-type variable | 1.012 ± 0.005 | 1.012 ± 0.006 | 0.883 | - | - | - | - | - |
|  | pH | Real-type variable | 6.389 ± 0.601 | 6.121 ± 0.795 | 0.219 | - | - | - | - | - |
|  | Proteins | Graded variable^4^ | 1.00 ± 1.50 | 1.27 ± 2.05 | 0.778 | - | - | - | - | - |
|  | Glucose | Graded variable^5^ | 0.00 ± 0.00 | 0.71 ± 2.02 | 0.213 | - | - | - | - | - |
|  | Ketones | Graded variable^6^ | 0.00 ± 0.00 | 0.01 ± 0.08 | 0.812 | - | - | - | - | - |
|  | Hemoglobin | Graded variable^7^ | 1.11 ± 1.76 | 1.52 ± 1.89 | 0.365 | - | - | - | - | - |
|  | Nitrite | Graded variable^8^ | 0.33 ± 1.00 | 1.27 ± 1.40 | 0.055 | - | - | - | - | - |
|  | Leukocytes esterase | Graded variable^9^ | 3.00 ± 1.22 | 2.59 ± 1.42 | 0.395 | - | - | - | - | - |
| ^1^ 0 for male, 1 for female. ^2^ <10^4/mL to ≥10^7/mL was assigned for rank 0 to 4. ^3^ <5/HPF to ≥100/HPF was assigned for rank 0 to 6. ^4^ Negative to (4+) >1000 mg/dL was assigned for rank 0 to 10. ^5^ Negative to (4+) >1000 mg/dL was assigned for rank 0 to 9. ^6^ Negative to (1+) 20 mg/dL was assigned for rank 0 to 2. ^7^ Negative to (3+)>1.0 mg/dL was assigned for rank 0 to 7.  ^8^ Negative to (3+) was assigned for rank 0 to 4. ^9^ Negative to (4+) 500/µL was assigned for rank 0 to 4. | | | | | | | | | | |
|  |  |  |  |  |  |  |  |  |  |  |
|  |  |  |  |  |  |  |  |  |  |  |
